# Engineered Bacteria EcN-MT Alleviate Liver Injury in Cadmium-Exposed Mice *via* its Probiotics Characteristics and Expressing of Metallothionein

**DOI:** 10.3389/fphar.2022.857869

**Published:** 2022-02-24

**Authors:** Changwei Zou, Ying Chen, Hongyu Li, Wenyu Li, Jin Wei, Ziyan Li, Xinliang Wang, Tingtao Chen, Hong Huang

**Affiliations:** ^1^ Key Laboratory of Poyang Lake Environment and Resource Utilization, School of Resources Environmental and Chemical Engineering, Ministry of Education, Nanchang University, Nanchang, China; ^2^ Queen Mary School, Nanchang University, Nanchang, China; ^3^ National Engineering Research Center for Bioengineering Drugs and the Technologies, Institute of Translational Medicine, Nanchang University, Nanchang, China

**Keywords:** cadmium, EcN-MT, metallothionein, inflammation, oxidative stress, intestinal microbiota

## Abstract

Cadmium (Cd) exposure is a widespread problem in many parts of the world, but effective means to treat Cd exposure is still lacking. Hence, an engineered strain expressing metallothionein (MT) named *Escherichia coli* Nissle 1917 (EcN)-MT was constructed, and its potential in the treatment of Cd exposure was evaluated. The *in vitro* studies showed that metallothionein expressed by EcN-MT could significantly bind Cd. Further, the *in vivo* results indicated that EcN-MT strain could reduce 26.3% Cd in the liver and increase 24.7% Cd in the feces, which greatly decreased malondialdehyde (MDA) levels and increased catalase (CAT), glutathione (GSH), and superoxide dismutase (SOD) levels in liver, and reduced the expression of toll-like receptor4 (TLR4), nuclear factor-κB (NF-κB), the myeloid differentiation factor 88 (Myd88) andincreased B-cell lymphoma 2 (Bcl-2)/Bcl-2-Associated X (Bax). Moreover, high throughput sequencing results indicated that EcN-MT strain greatly enhanced the beneficial bacteria of Ruminococcaceae, Lactobacillaceae, *Akkermansia*, *Muribaculaceae*, *Lachnospiraceae*, *Dubosiella* and restored the disturbed microbial ecology to the normal level. Therefore, the high Cd binding capacity of the expressed metallothionein, together with the beneficial characteristics of the host bacteria EcN, makes EcN-MT a sound reagent for the treatment of subchronic Cd exposure-induced liver injury.

## Introduction

Heavy metal pollution is the pollution caused by the entry of some biotoxic metals and metalloids and their compounds into the environment, which causes widespread concern due to its high toxicity, difficulty in being degraded, biomagnification and bioaccumulation along the food chain (Ali et al., 2019). It is reported that in China alone, contamination by cadmium (Cd), arsenic and lead has led to an annual loss of about 20 billion RMB in agricultural production and about 12 million tons of contaminated food (Clemens et al., 2013). Contaminated food and water are major sources of heavy metal exposure for non-occupational populations. Studies have shown that heavy metal exposure has toxic effects on liver, kidney, neurological, cardiovascular, and pulmonary fibrosis diseases (Hyder et al., 2013; Wang et al., 2021). In particular, Cd pollution is one of the most serious heavy metal pollutions in China, and with the development of industry, it is expected that the risk of Cd exposure to human health will further increase in the next decade (Baba et al., 2013; Xu et al., 2021).

Currently, the two main types of methods used to treat heavy metal exposure are chelation and antioxidants. However, both therapeutic options present certain limitations. Chelating agents are generally effective only for a short period of time, and can cause liver injury when used at doses higher than 1/4 LD_50_ (Nordberg, 1984). Antioxidants mitigate the oxidative stress caused by heavy metal exposure, but have been reported unable to reduce heavy metals through chelation or excretion (Eybl et al., 2006). These limitations have prompted investigators to seek for more effective solutions involving other mechanism pathways. In addition to external therapies such as chelation and antioxidants, the body has its own removal mechanism for heavy metal exposure, namely, chelation of excess heavy metals by metallothionein. Metallothioneins (MT) are a class of low molecular weight, homocysteine proteins found in most eukaryotic organisms that bind to heavy metals primarily through cysteine thiol groups to form non-toxic chelates (Andreani et al., 2011; Shen et al., 2019). Meanwhile, MT is one of the most powerful endogenous free radical scavengers known. The researchers found that MT gene deletion exacerbated the injury caused by heavy metal exposure, further confirming its important role in the protection of the organism (Liu et al., 2002). The study also showed that exogenous MT not only helped to eliminate heavy metal Cd in aquatic animals but also reduced liver injury caused by thallium poisoning and to some extent reduced inflammation and collagen deposition, thus alleviating pulmonary fibrosis (Helal and Helal, 2009; Kilic and Kutlu, 2010; Duan et al., 2018).

Increasing evidence underscore the close association of gut microbes with numerous diseases such as cirrhosis, alcoholic fatty liver, and nonalcoholic fatty liver (Zhang et al., 2015; Schwabe and Greten, 2020). The gut microbiota is regarded as a potential therapeutic target by degrading other potentially toxic dietary products or producing nutritional metabolites (Dapito et al., 2012). Probiotics are living microorganisms that have been widely recognized for their important contribution to the regulation of intestinal microbiota (de Vrese and Schrezenmeir, 2008), and some recent reports indicated that probiotics can regulate disturbances of intestinal microbiota caused by the exposure to heavy metals (Zhai et al., 2017; Zhang et al., 2018). Among all the probiotic strains, *Escherichia coli* Nissle 1917 (EcN) has received much attention. Since its discovery in 1957, EcN has played an important role as a Gram-negative probiotics in regulating the intestinal microbiota and suppressing enteritis (Sassone-Corsi et al., 2016), due to its excellent safety profile, good tolerability, clear genetic background, availability of genetic manipulation, and excellent colonization properties (Westendorf et al., 2005; Luo et al., 2020). Moreover, with the boom of synthetic biology, EcN has been used as a engineered strain vector for the treatment of phenylketonuria in a phase I clinical study (Puurunen et al., 2021).

In this study, the MT gene was integrated into plasmid pET-28a, which was then transferred into EcN to construct the engineered bacterium EcN-MT to continuously express MT. Thus, it is expected to have the effect of one plus one more than two. The treatment effect of EcN-MT on Cd was studied using a subchronic Cd exposure mice model, which may provide a basis for its potential use in clinic.

## Materials and Methods

### Strain Construction and Evaluation *in vitro*

MT genes (gene ID 856450) were inserted into the pET-28a plasmid, and then was heat stimulated into the receptor *E. coli* Nissle1917 to construct the engineered bacteria EcN-MT. Then, the growth curves (Luo et al., 2020), plasmid stability (Liu et al., 2019), acid resistance, bile salt resistance and oxidation resistance capability (Xia et al., 2020; Wang et al., 2020) were evaluated.

Finally, the binding capability of EcN-MT and EcN to Cd was tested. Briefly, EcN-MT and EcN were respectively co-incubated with 0.545 mM of CdCl_2_ for 1 h, centrifuged at 5,000 × g for 5 min, and the supernatant was treated according to the water quality—32 elements determination—inductively coupled plasma emission spectrometry (HJ776-2015) and detected by ICP-AES for Cd content (Alexander et al., 2017).

### Development and Treatment of Cadmium Exposure Model

Six-week-old Male (Sogawa et al., 2001) C57BL/6J mice (18–20 g), provided by Hunan SJA Laboratory Animal, were maintained in the specific pathogen free (SPF) laboratory animal barrier system of the Institute of Translational Medicine of Nanchang University under standard conditions (humidity 40–70%, temperature 20–26°C, 12/12 light-dark cycle) and were fed with standard mice maintain diet (Xie tong biological, CN, Cat# 101139). After 1 week of initial adaptation to the cage food and laboratory conditions, a total of 50 mice were randomly divided into groups C (*n* = 10, drank distilled water, gavaged with gelatin saline per day), M (*n* = 10, treated with 0.545 mM cadmium chloride (CdCl_2_) (Thijssen et al., 2007; Zhai et al., 2014) (aladdin, CN, Cat#C116342), gavaged with gelatin saline per day), EcN (*n* = 10, treated with 0.545 mM CdCl_2_, gavaged with 10^9^ CFU EcN per day), MT (*n* = 10, treated with 0.545 mM CdCl_2_, gavaged with 2 mg/kg body weight MT (Yuanye biological, CN, Cat#S12070) per day), and EcN-MT (*n* = 10, treated with 0.545 mM CdCl_2_, gavaged with 10^9^ CFU EcN-MT per day). After 8 weeks the mice were euthanized by a skilled technician using an intraperitoneal injection of 1% sodium pentobarbital (40 mg/kg) followed by assisted decervicalization. To further explore the mechanism of EcN-MT, 40 six-week-old male C57BL/6J mice were purchased for the experiment. Then, after 1 week of adaptive feeding, 40 mice were randomly divided into four groups: group C (*n* = 10, drank distilled water, gavaged with gelatin saline per day), group M (*n* = 10, treated with 0.545 mM cadmium chloride (CdCl_2_) (aladdin, CN, Cat#C116342), gavaged with gelatin saline per day), group EcN-MT (*n* = 10, treated with 0.545 mM CdCl_2_, gavaged with 10^9^ CFU EcN-MT per day), and group PDTC [treated with 0.545 mM CdCl_2_, Intraperitoneal injection 50 mg/kg body weight Pyrrolidinedithiocarbamic acid (PDTC) (MedChemExpress, Cat#HY-18738)]. The mice were euthanized in the same manner as above at the end of the eight-week experiment.

### Estimation of Cadmium in Liver Tissue and Feces

Weigh 0.1 g of mice liver tissue and feces respectively and homogenize in 1 ml of PBS. Then, the samples were digested overnight in 3 ml of concentrated nitric acid (65% (v/v)) and transferred to a Teflon digestion tube. After the first digestion at 80°C for 1 hour, 2 ml of H_2_O_2_ [30% (v/v)] was added and the samples were digested at 120°C until they were completely transferred into a clear colorless liquid, then filtered through a 0.22 μm hydrophilic PTFE membrane filter and diluted with MilliQ water containing 2% HCl and Indium (In) elements (50 ug/kg). The prepared samples were analyzed directly by ICP-MS and Cd calibration curves. The Cd concentrations were determined by plotting the calibration curves of ICP-MS Cd standards with known concentrations and the internal standard element In concentration.

### Histology and Histopathology

The liver tissues of euthanized mice were dissected and then randomly selected, fixed with 4% paraformaldehyde and embedded in paraffin. Next, the embedded tissues were cut into 2–4 μm serial sections and after dewaxing, staining, dehydration and transparency treatment, the hematoxylin eosin-stained liver tissues could be sealed for observation under light microscope.

Then, the colon tissues of mice were randomly selected for rinsing, fixation, immersion wax embedding, dehydration, transparency, dehairing, hematoxylin eosin staining, and finally histological sections were examined by light microscopy. The histological injury was evaluated by a semiquantitative method and scored on a scale of 0–4 essentially as described previously (Cao et al., 2017).

### Western Blotting

Wet protein blotting was performed as described previously (Xia et al., 2020). Briefly, 1 ml of RIPA lysis buffer and a corresponding dose of mixed protease inhibitor were added per 0.1 g of tissue to obtain the cell lysis supernatant. Then it was measured by BCA method at 592 nm for protein concentration, mixed with 5× protein loading buffer and water bath at 100°C for 5–10 min until denaturation. After that, the proteins were transferred from the polyacrylamide-SDS gels to polyvinylidene fluoride (PVDF) in TBS containing 0.1% Tween-20, 5% nonfat dry milk for 1 h. Next, the bands were incubated overnight at 4°C with anti-Bax (Cat#5174), anti-Bcl2 (Cat#5174), anti-TLR4 (Cat#19811-1-AP), anti-MyD88 (Cat#66660-1-Ig), anti-p65 (Cat#10745-1-AP), anti-p-p65 (Cat#AF 2006), anti-a-SMA (Cat#14395-1-AP), anti-Occludin (Cat#27260-1-AP), anti-β-actin (Cat#66009-1-Ig). Afterward the bands were conjugated with secondary antibodies corresponding to horseradish peroxidase, and finally the band strength was shown by chemiluminescence. Specific antibody information is shown in Supplementary Table S1.

### Measurement of Oxidation-Related Biomarkers

According to the instructions of the kit, 0.1 g of liver tissue was weighed and added to 1 ml of the extraction solution provided in the kit, homogenized in an ice bath, centrifuged at 8,000 g for 10 min at 4°C, and the supernatant was obtained. And it was measured by enzyme standard according to the principle that malondialdehyde (MDA) (Suzhou comin Biotechnology co., Ltd., CN, Cat#MDA-1-Y) could react with thiobarbituric acid to form a red product with maximum absorbance at 532 nm, catalase (CAT) (Suzhou comin Biotechnology co., Ltd., CN, Cat#GSH-1-W) activity could be measured by reduced hydrogen peroxide at 240 nm, glutathione (GSH) (Suzhou comin Biotechnology co., Ltd., CN, Cat#GSH-1-W) could react with DTNB to form a complex with a characteristic peak at 412 nm, Superoxide dismutase (SOD) (Suzhou comin Biotechnology co., Ltd., CN, Cat#SOD-1-Y) could scavenge superoxide anion (O^2-^) and thus reduced methanogenesis at 560 nm.

### Real-Time PCR

After fast isolation of total RNA from fresh hepatocytes using Trizol, complementary first-strand cDNA synthesis was performed with the Prime Script RT Master Mix Reverse Transcription Kit (Prime Script RT Master Mix; Ta Ka Ra Biotechnology). Next, SYBR green method with ABI 7900HT fast real-time PCR system was used to detect the expression of Interleukin (IL)-1β, IL-6, and tumour necrosis factor (TNF)-α inflammatory factor markers. The primers for these analyses are listed in Supplementary Table S2. In the end, Real-time qPCR reactions were performed in triplicate using GAPDH as the internal reference gene, and calculated using the 2^−ΔΔCt^ method for analysis.

### 16S rRNA Gene Sequencing

According to references (Tao et al., 2020; Wang and Liu, 2020), bacterial genomic DNA was extracted and the target fragment of the 16S rRNA V4 region was amplified using bacterial universal primer 520F (5*′*-AYTGGGYDTAAAGNG-3′) and 802R (5′-TACNVGGGTATCTAATCC-3′). Next, amplification products were then double-ended (paired-end) sequenced against the colony DNA fragment using the Illumina platform. Then, the ASV/OTU signature sequences were obtained using the DADA2 method of analysis software for quality control. Afterward data processing was performed using QIIME v. 1.9.180 to multiplex the raw fastq data and high quality reads were obtained by quality filtering parameters (Phred quality score ≥ 20, minimum read length = 75% of nucleotides of 250). Whereafter, cluster 16 S rRNA gene sequences were then read into OTUs using UCLUST81 and the Greengenes reference database v13.882,83, and species-level taxonomic assignments were then obtained using Megablast84 with the reference sequences of candidate OTUs from the Greengenes database. Finally, sequences were used to compare the relative abundance of OTUs in at least five samples for analyses such as species composition, alpha diversity, and beta diversity.

### Statistical Analysis

The data were analyzed with Prism8 (GraphPad). Two groups of data were compared using Student’s t-test. Three and multiple groups of data were analyzed using two-way ANOVA and multiple comparisons to detect statistical differences. Data for all experimental outcomes were expressed as mean plus or minus standard deviation.

## Results

### Evaluation of Probiotic Characteristics of EcN-MT *in vitro*

**FIGURE 1:**
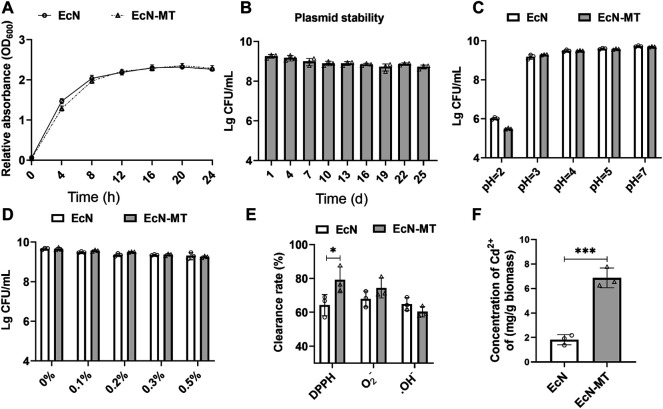
Evaluation of the probiotic characteristics of EcN-MT. Values are presented as means ± SD (*n* = 3). **(A)** Growth curves of EcN and EcN-MT. **(B)** Plasmid stability test of EcN-MT. **(C)** The acid tolerance of EcN and EcN-MT. **(D)** The cholate tolerance of EcN and EcN-MT. **(E)** The antioxidant ability of EcN and EcN-MT. **(F)** The ability of EcN and EcN-MT to bind cadmium ions. *p < 0.05.

Growth curve assay was used to examine the growth characteristics of EcN-MT and the results showed no difference in growth characteristics between EcN-MT and EcN strains (Figure 1A). Subsequently, ELISA assay was used to examine the ability of EcN-MT to express MT (Supplementary Figure S1). The plasmid stability of EcN-MT was then assessed and suggested that viable EcN-MT still reached 5 × 10^8^ CFU/ml after 25 days of passaging once per day (Figure 1B). In addition, the resistance of EcN and EcN-MT to high concentrations of acid and bile salts was evaluated separately, and the results showed that both EcN and EcN-MT had good resistance to acid and bile salts (Figures 1C,D). This indicated that both EcN-MT and EcN had the ability to resist gastric acid. Finally, antioxidant properties and heavy metal binding capacity were evaluated. In the antioxidant assay, EcN and EcN-MT showed good antioxidant capacity, especially in DPPH reducing capacity, with EcN-MT scavenging capacity being superior to EcN (Figure 1E, *p* < 0.05). In the heavy metal binding capacity test, EcN-MT could bind the heavy metal Cd to a greater extent compared to EcN (6.536 vs. 1.822 mg/g biomass) (Figure 1F), suggested that EcN-MT has the potential to treat Cdexposure.

### EcN-MT Treatment Ameliorates Liver Injury, Fibrosis, Oxidative Stress in the Liver of Mice

**FIGURE 2:**
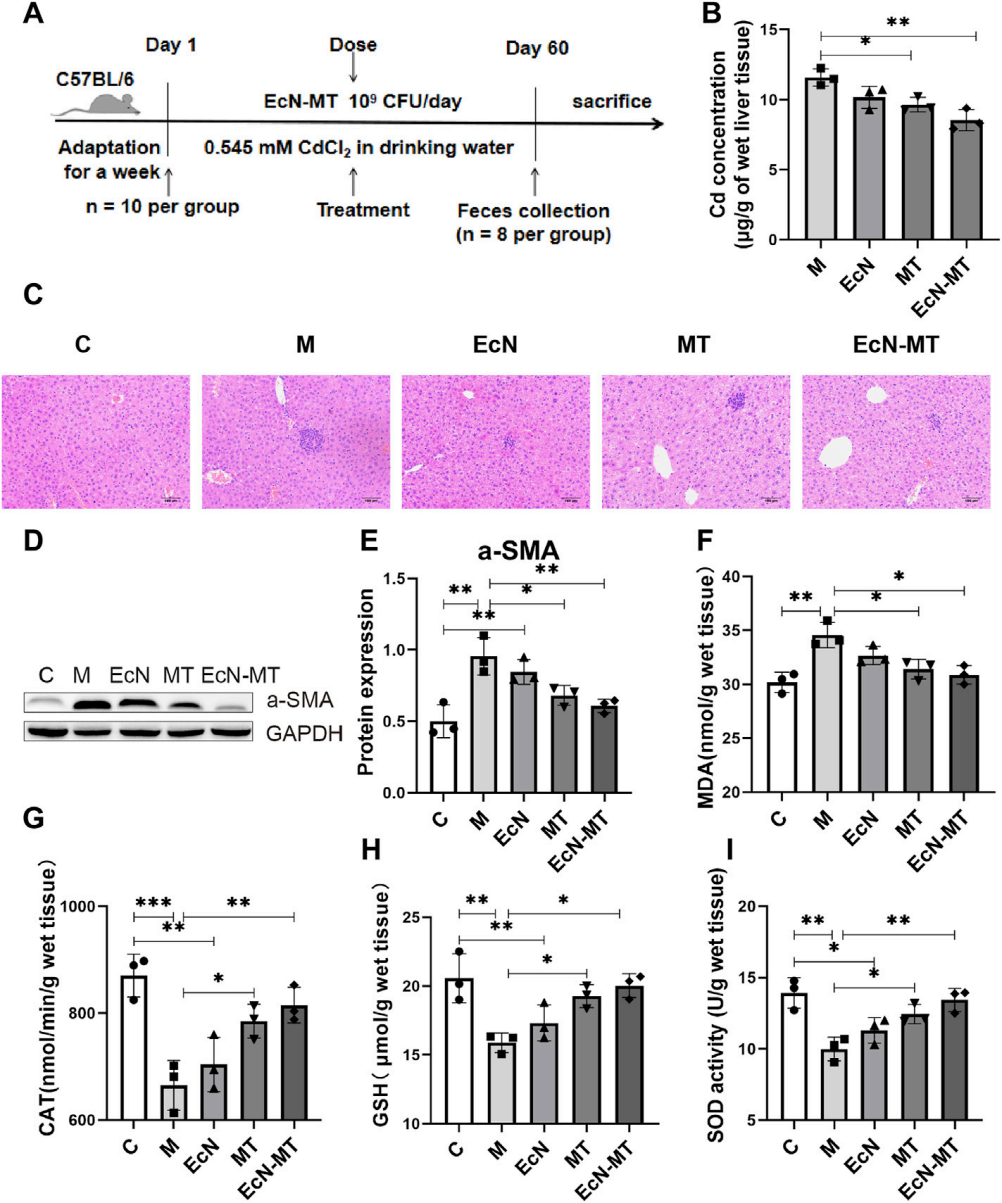
EcN-MT treatment reduce liver injury, fibrosis, oxidative stress in the liver of mice. Values are presented as means ± SD (*n* = 3). **(A)** Treatment of EcN-MT in mice with subchronic Cd exposure. **(B)** Cd concentrations in the liver assayed by ICP-MS. **(C)** HE staining image of liver tissue (200×). **(D)** Western bloting analysis of a-SMA expression in liver tissues. **(E)** The relative expressions of a-SMA were quantified by ImageJ. GAPDH was used as an internal control. The activity of **(F)** MDA, **(G)** CAT, **(H)** GSH, **(I)** SOD. *p < 0.05, **p < 0.01, ***p < 0.001.

Cd-induced subchronic liver injury model was used to investigate the effect of EcN-MT on liver injury induced by Cd exposure (Figure 2A). The results of heavy metal Cd assay in liver tissues showed that the Cd concentration in liver tissue was significantly higher in group M compared with group C. Cd levels were slightly decreased after EcN treatment compared with the M group; in contrast, significant levels of Cd in liver tissue were reduced after MT and EcN-MT treatment (MT vs. M, *p* < 0.05; EcN-MT vs. M, *p* < 0.01) (Figure 2B). The results of group C were not shown in Figure 2B due to the very low Cd concentration in the liver tissue (mean level was only 0.0242 mg/g wet weight). Then detection results of heavy metal cadmium in feces showed that MT and EcN-MT treatment, compared with group M, significantly increased the fecal Cd levels in mice (MT vs. M, *p* < 0.05; EcN-MT vs. M, *p* < 0.01) (Supplementary Figure S2). Next, hematoxylin-eosin (HE) staining showed that group M had a large number of eosinophil aggregates and some degree of focal necrosis of hepatocytes, while liver inflammation was reduced after treatment in EcN, MT and EcN-MT groups, with only mild cell swelling and inflammatory infiltration in the EcN-MT group presenting the most significant efficacy (Figure 2C). Meanwhile a-SMA protein expression results as shown in Figure 2D indicated that its expression level was significantly upregulated by Cd exposure, but was decreased after MT and EcN-MT treatment (MT vs. M, *p* < 0.05; EcN-MT vs. M, *p* < 0.05) (Figure 2E). In addition, the results of oxidative stress in the liver showed that MDA levels were significantly increased and CAT, GSH, and SOD levels were significantly decreased in the M group, while MT and EcN-MT treatments reversed these alterations (Figures 2F–I, *p* < 0.05).

### EcN-MT Reduces Liver Inflammation and Apoptosis

**FIGURE 3:**
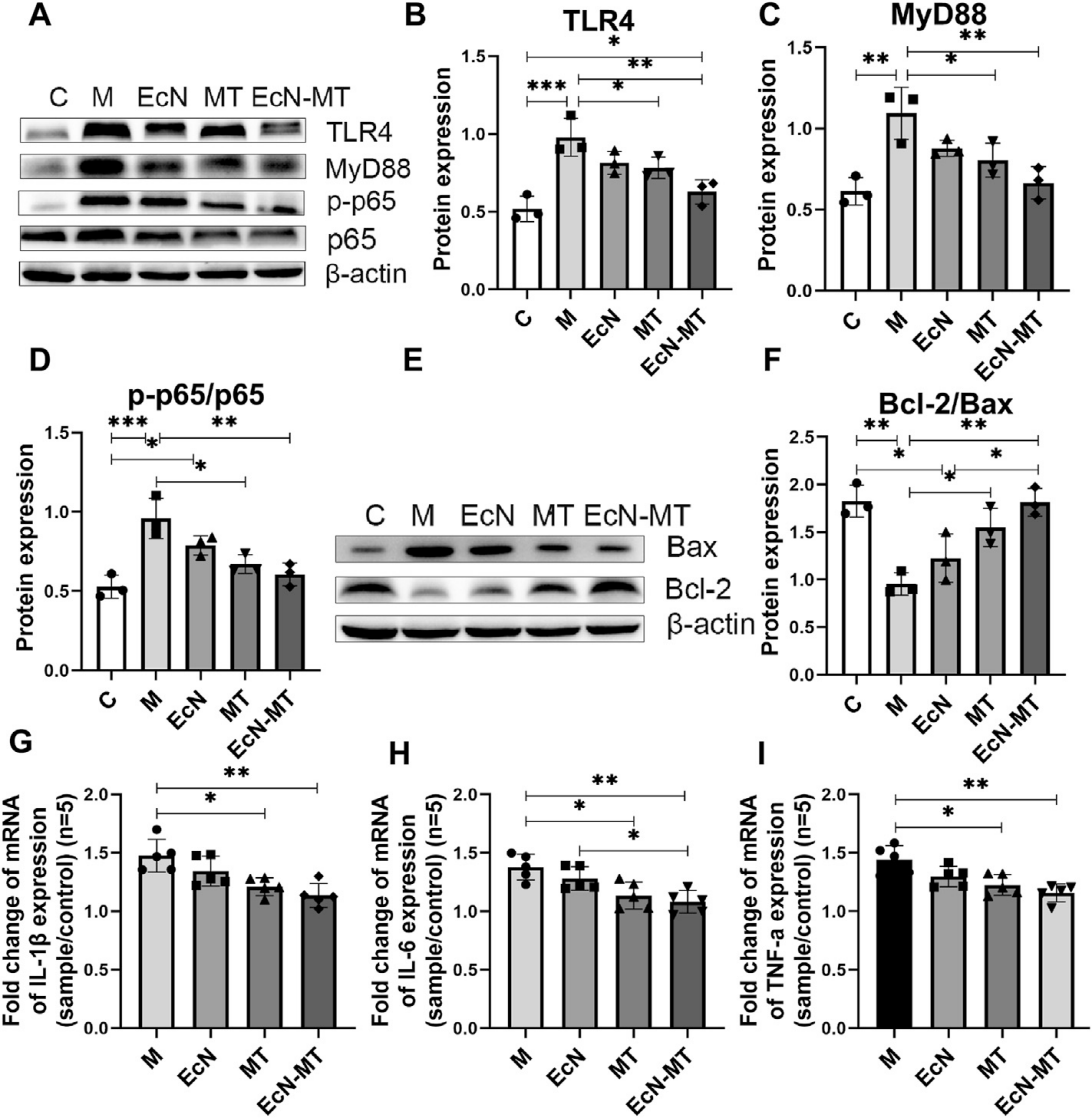
EcN-MT reduced liver inflammation and apoptosis. Values are presented as means ± SD (*n* = 3, except q-PCR results repeated five times). **(A)** Western bloting analysis of TLR4, MyD88, p-p65, p65 expression in liver tissues. The relative expressions of **(B)** TLR4, **(C)** MyD88, **(D)** p-p65/p65 were quantified by ImageJ. β-actin was used as an internal control. **(E)** Western bloting analysis of Bax, Bcl-2 expression in liver tissues. **(F)** The relative expressions of Bcl-2/Bax were quantified by ImageJ. β-actin was used as an internal control. The relative mRNA expressions of **(G)** IL-1β, **(H)** IL-6, **(I)** TNF-α in liver tissues were detected by q-PCR. *p < 0.05, **p < 0.01, ***p < 0.001.

To explore the potential mechanisms by which EcN-MT treatment attenuates subchronic Cd exposure-induced liver injury, key proteins of the inflammatory pathway (TLR4 signaling) and apoptotic pathway (Bcl-2 family proteins) were investigated. As shown in Figures 3A–D, oral administration of CdCl_2_ significantly increased the expression of TLR4 (M vs. C, *p* < 0.001), MyD88 (M vs. C, *p* < 0.01), and p-p65/p65 (M vs. C, *p* < 0.001) compared with the C group. TLR4 (0.783, 0.628, respectively), MyD88 (0.805, 0.666, respectively) and p-p65/p65 (0.671, 0.605, respectively) were significantly decreased in the MT and EcN-MT groups compared with the M group, respectively. Meanwhile, Bcl-2/Bax expression was significantly reduced in the M group compared with the C group (M vs. C, 0.954 vs. 1.826), while MT and EcN-MT treatments reversed the reduction in Bcl-2/Bax expression induced by Cd exposure (MT vs. M, 1.549 vs. 0.954, EcN-MT vs. M, 1.814 vs. 0.954) (Figures 3E,F). In addition, the results of the relative expression of inflammatory factors revealed that the expression of IL-1β, IL-6 and TNF-α was significantly increased in the M group compared with the C group, while the relative expression of IL-1β (1.210, 1.136, respectively), IL-6 (1.135, 1.082, respectively) and TNF-α (1.226, 1.159, respectively) was significantly reduced in the livers of the MT and EcN-MT groups compared with the M group (Figures 3G–I; *p* < 0.05).

### EcN-MT Improved Intestinal Microbiota in Subchronic Cd -Exposed Mice

**FIGURE 4:**
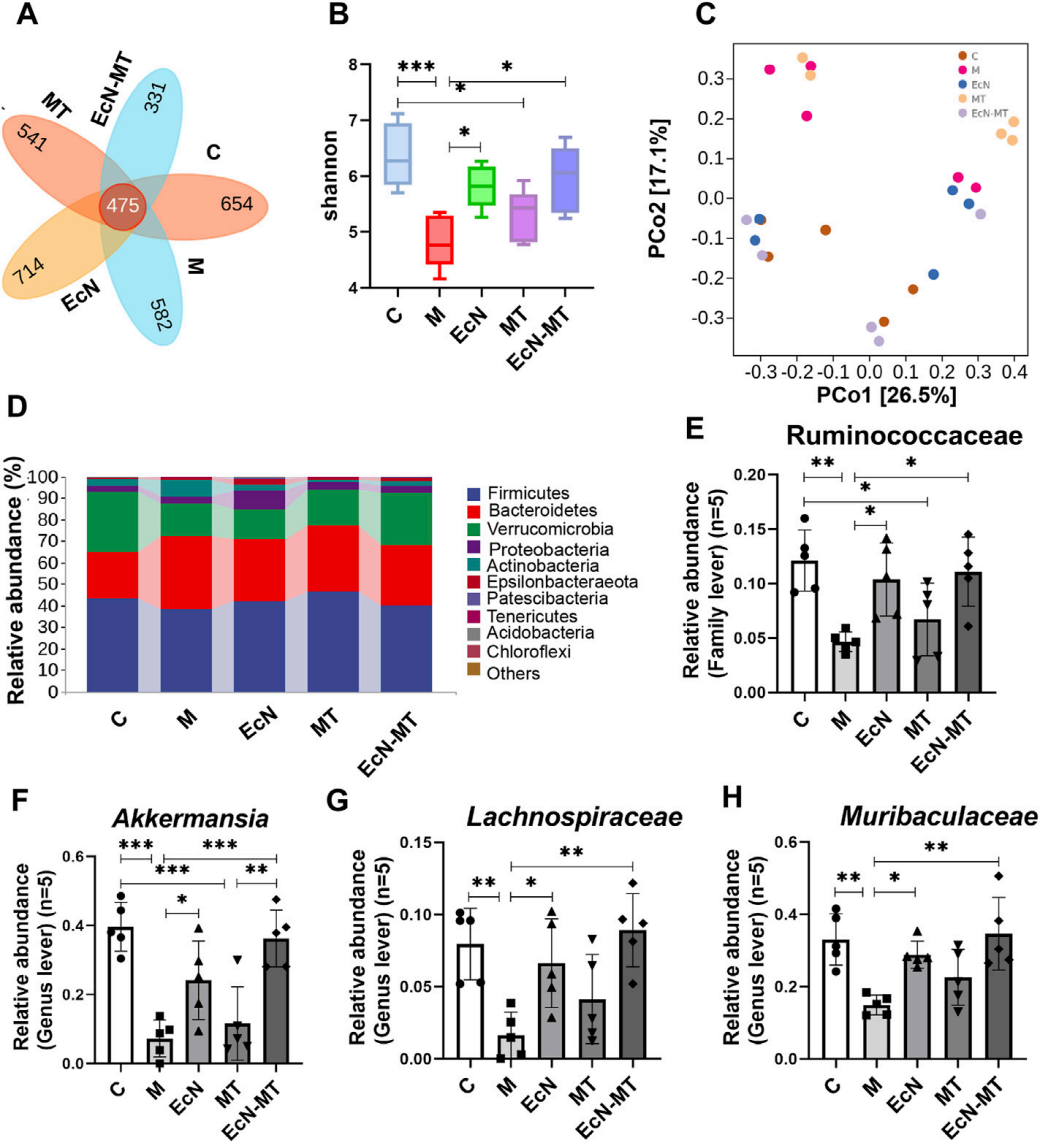
EcN-MT improved intestinal microbiota in subchronic Cd-exposed mice. Values are presented as means ± SD (*n* = 5). **(A)** Venn map representation of OTUs. **(B)** The Shannon index. **(C)** PCoA of β diversity index. **(D)** Microbial composition at the phyla level. The relative abundance of **(E)** Ruminococcaceae, **(F)** *Akkermansia*, **(G)** *Muribaculaceae*, **(H)** *Lachnospiraceae* were analyzed. *p < 0.05, **p < 0.01, ***p < 0.001.

High-throughput sequencing methods were used to study the effects of EcN, MT and EcN-MT on the intestinal microbiota of mice with subchronic Cd exposure. The Venn method analyze indicated that 475 common OTUs were identified from each group, and the number of unique OTUs in C, M, EcN, MT and EcN-MT groups were 654, 582, 714, 541 and 331, respectively (Figure 4A). The results of Shannon index of α diversity showed that compared with C group, the community diversity of M group was decreased, and EcN, MT and EcN-MT treatment alleviated the change, among which EcN-MT treatment had the most significant effect (M vs. EcN-MT, 4.835 vs. 5.948) (Figure 4B). And then the results of PCoA showed that the M and MT groups had similar sample points and were farther from the C group in the plot, indicating that the microbial diversity of the M and MT groups was significantly different from that of the C group. Meanwhile, samples from the EcN-MT and EcN groups in the plot were similar to the C group and far from the M group, indicated that the microbial diversity of the EcN and EcN-MT treatments was significantly different from that of the M group (Figure 4C). In addition, the results of species composition analysis at the phylum level showed a significant decrease in the thick-walled phylum and an increase in the bacillus-like phylum in the M group compared with the C group. However, the EcN and EcN-MT treatments increased the abundance of the thick-walled phylum and decreased the abundance of the anaphyla phylum compared to the M group. (Figure 4D). Subsequently, the data of the top 10 abundant microbial populations at family level and genus level were analyzed and the results showed that compared to C group, M group presented a significant reduction in the abundance of Ruminococcaceae at family level, *Akkermansia, Muribaculaceae, Lachnospiraceae* at genus level. After MT treatment, the abundance increased but were not significantly different, but EcN and EcN-MT treatments decreased the alteration presented in the M group (Figures 4E–H, *p* < 0.05).

### Suppression of NF-κB Activation Reduced Hepatic Inflammation and Oxidative Stress

**FIGURE 5:**
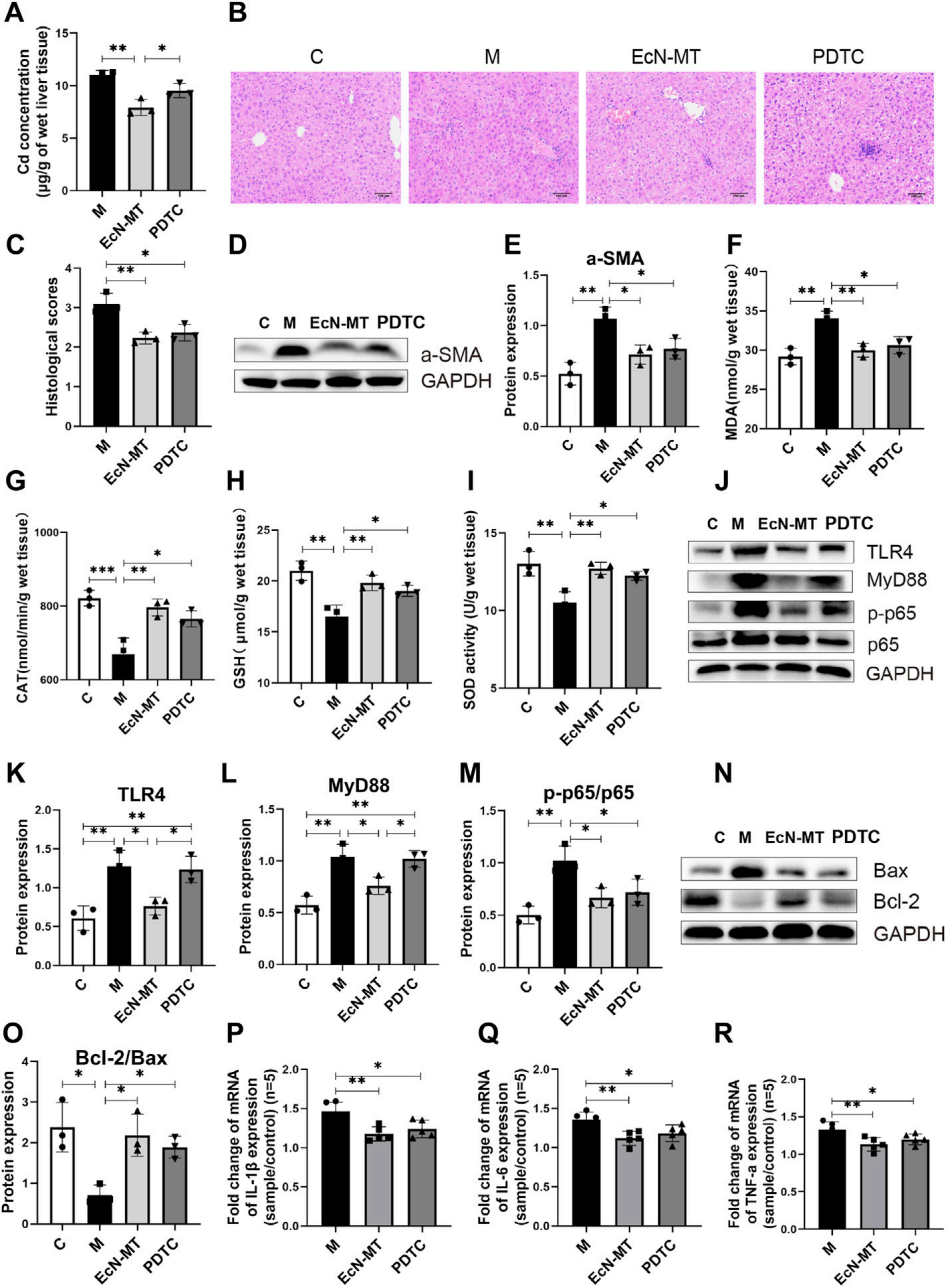
Suppression of NF-κB activation reduced hepatic inflammation and oxidative stress. Values are presented as means ± SD (*n* = 3, except q-PCR results repeated five times). **(A)** Cd concentrations in the liver assayed by ICP-MS. **(B)** HE staining image of liver tissue (200×). **(C)** Histological score of liver injury. After 8 weeks of exposure to drinking water, the pathological changes of liver tissues stimulated by cadmium chloride (0.545 mM) were determined. **(D)** Western bloting analysis of a-SMA expression in liver tissues. **(E)** The relative expressions of a-SMA were quantified by ImageJ, expressions of a-SMA were quantified by ImageJ. GAPDH was used as an internal control. The activity of MDA; CAT; GSH; SOD, activity of **(F)** MDA; **(G)** CAT; **(H)** GSH; **(I)** SOD. **(J)** Western bloting analysis of TLR4, MyD88, p-p65, p65 expression in liver tissues. The relative expressions of **(K)** TLR4, **(L)** MyD88, **(M)** p-p65/p65 were quantified by ImageJ. GAPDH was used as an internal control. **(N)** Western bloting analysis of Bax, Bcl-2 expression in liver tissues. **(O)** The relative expressions of Bcl-2/Bax were quantified by ImageJ. GAPDH was used as an internal control. The relative mRNA expressions of **(P)** IL-1β, **(Q)** IL-6, **(R)** TNF-α in liver tissues were detected by q-PCR. *p < 0.05, **p < 0.01, ***p < 0.001.

To further determine whether EcN-MT attenuated liver injury by inhibiting the activation of TLR4/MyD88/NF-κB signaling pathway, NF-κB inhibitor (PDTC) was used in a subchronic Cd exposure mice model. Notably, the results of heavy metal Cd assay in liver tissues showed that regarding the ability to reduce accumulated Cd in liver tissues, PDTC treatment was not as effective as EcN-MT (EcN-MT Vs PDTC, 7.925 vs. 9.547) (Figure 5A, *p* < 0.05). Meanwhile, the results of the determination of heavy metal Cd in feces showed that the Cd content in the feces of mice increased significantly after EcN-MT treatment, however, there was no change in PDTC treatment (EcN-MT vs. PDTC, *p* < 0.05) (Supplementary Figure S3). Subsequent HE staining showed the same hepatocellular edema and reduced central venous congestion after PDTC and EcN-MT treatment (Figures 5B,C, *p* < 0.05). Similarly, PDTC treatment and EcN-MT treatment significantly reversed the upregulated a-SMA protein, increaseing MDA, decreaseing CAT, GSH and SOD induced by Cd exposure (Figures 5D–I, *p* < 0.05). Furthermore, the results of key protein expression assays of inflammatory pathways (TLR4/NF-κB signaling) showed that PDTC treatment did not reverse the upregulation of TLR4 (EcN-MT vs. PDTC, 0.763 vs. 1.234) and MyD88 (EcN-MT vs. PDTC, 0.761 vs. 1.021) expression induced by cadmium exposure compared to the EcN-MT group whereas only p-p65/p65 expression was reduced. (Figures 5J–M, *p* < 0.05). Also, the results of key protein expression assays of Bcl-2 family proteins indicated that PDTC treatment as effective as EcN-MT treatment (Figure 5N, O, *p* < 0.05). Then, the results of inflammatory factor assay also showed that the upregulation of IL-1β, IL-6 and TNF-α expression induced by Cd exposure was significantly reduced by EcN-MT and PDTC treatment (Figures 5P–R, *p* < 0.05).

### EcN-MT Rather Than NF-κB Inhibitors Improved Intestinal Microbiota

**FIGURE 6:**
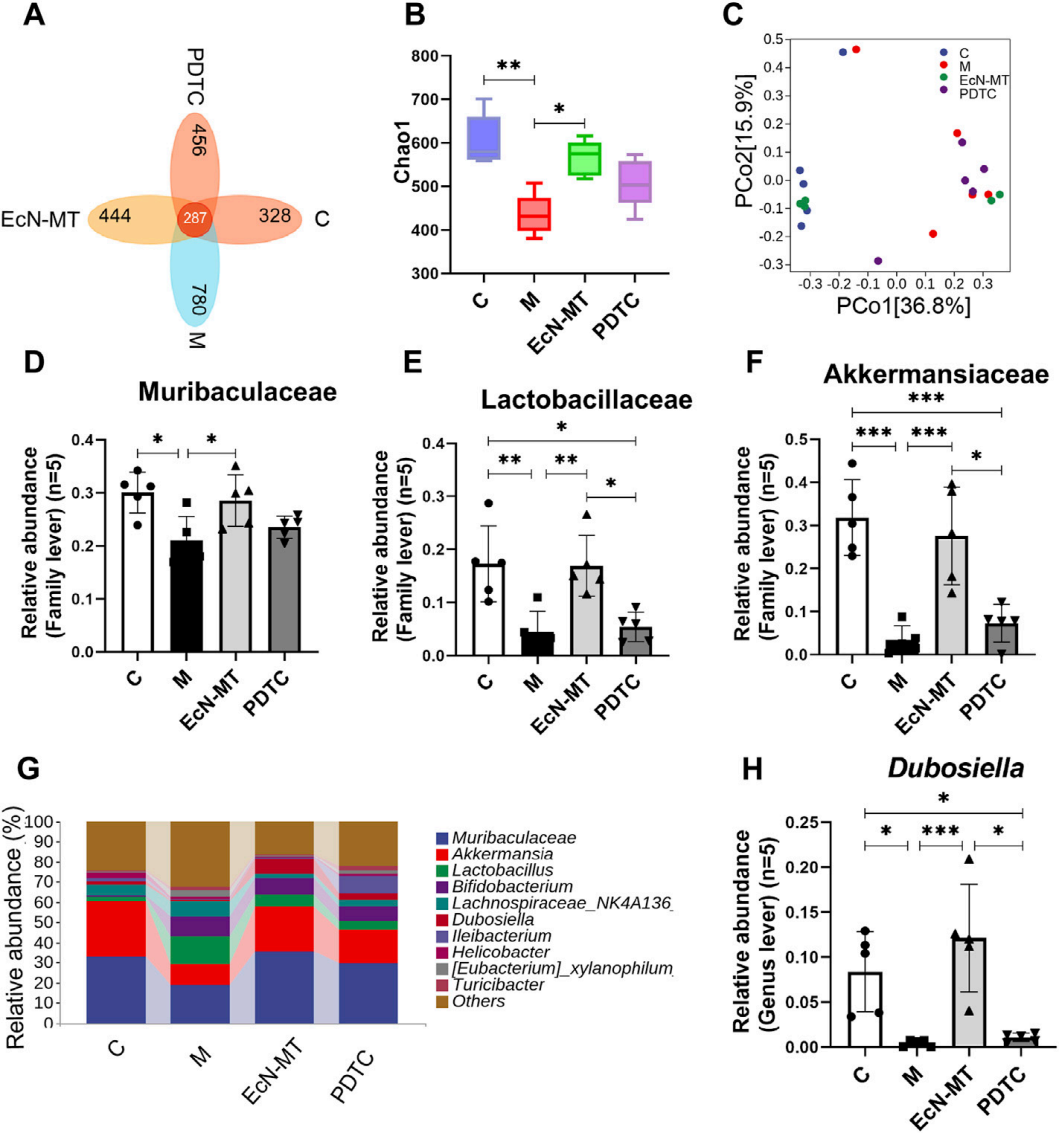
EcN-MT rather than NF-κB inhibitors improved intestinal microbiota. Values are presented as means ± SD (*n* = 5). **(A)** Venn map representation of OTUs. **(B)** The Chao1index. **(C)** PCoA of β diversity index. **(D)** Microbiota composition at the genus level. The relative abundance of **(E)** Akkermansia, **(F)** Muribaculaceae, **(G)** Lachnospiraceae **(H)** Dubosiella were analyzed. *p < 0.05, **p < 0.01, ***p < 0.001.

16S rRNA was used to study the effect of NF-κB inhibitors on the intestinal microbiota of Cd-exposed mice. Then analysis by the Venn method showed that 287 common OTUs were identified from each group, with the number of unique OTUs in C, M, EcN-MT and PDTC being 328, 780, 444 and 456, respectively (Figure 6A). Remarkably, the results of the chao1 index of α diversity showed no significant change in community diversity after the PDTC treatment compared to the EcN-MT treatment (Figure 6B, *p* < 0.05). Meanwhile, the results of PCoA showed that the sample points in the PDTC group were not far from the M group compared to the EcN-MT treatment (Figure 6C). Furthermore, for the composition of the top 10 family-level microbiota, PDTC treatment was not shown to upregulate the abundance of Muribaculaceae, Akkermansia, and Lactobacillaceae compared to the EcN-MT group (Figures 6D–F, *p* < 0.05). Similarly, analysis of the top 10 genus-level microbial populations showed that PDTC treatment did not significantly increase the relative abundance of *Dubosiella* compared to the EcN-MT group (Figures 6G–I, *p* < 0.05).

## Discussion

Cd is known as a widespread environmental toxicant that contaminated water, food, etc., which made Cd exposure unavoidable. The liver is the main target organ for Cd accumulation and exposure (Uetani et al., 2006). Cd exposure could cause hepatic lesions, fibrosis inflammation and oxidative stress in liver tissues, which is one of the important mechanisms of Cd-induced liver injury (Saeedi et al., 2020; Xu et al., 2021). Although evidence so far have indicated that probiotic bacteria can protect against Cd-exposure hepatoexpoure (Zhai et al., 2014), the role of modified engineered bacteria in Cd-induced subchronic liver injury has rarely been reported.

Subchronic liver injury provides a more realistic simulation of human exposure to a contaminated environment, and there are limitations to the treatment of liver injury caused by subchronic Cd exposure. Therefore, in this study, we brought up a new therapeutic option for subchronic Cd exposure-induced liver injury, and carried out several experiments to verify its therapeutic functions and explore its potential mechanisms. *In vitro*, growth curve test, plasmid stability test and metallothionein expression test showed that EcN-MT was successfully constructed and had good plasmid stability and metallothionein expression ability. Then acid tolerance and bile salt tolerance tests showed that EcN-MT had the ability to tolerate gastric acid and the potential of oral administration. Subsequently, the results of antioxidant test showed that there was no significant difference between EcN-MT and EcN in OH^−^ radical and O_2_^−^ radical scavenging ability. However, EcN-MT was superior to EcN in DPPH radical scavenging capacity, indicating that EcN-MT had better antioxidant capacity. In addition, the heavy metal binding test showed that the heavy metal binding capacity of EcN-MT was better than that of EcN and reached 6.7 mg/g biomass. The above experimental results indicated that EcN-MT had the potential to treat Cd exposure (Figure 1, Supplementary Figure S1).

Then, subchronic Cd exposure animal models were used to test whether EcN-MT is efficacious *in vivo*. Consistent with the previous studies (Zhai et al., 2019), Cd exposure increased hepatic Cd accumulation, while EcN-MT supplementation significantly reduced Cd levels in the liver. This may be due to the binding properties of EcN-MT to the heavy metal Cd and because the expressed MT protein can chelate with excess free heavy metal ions (Huang et al., 2020), which promoted the excretion of Cd, leading to decrease Cd levels in the liver. The results of fecal Cd assay further confirmed this conjecture. Also, HE staining results and a-SMA assay showed that EcN-MT supplementation reduced liver inflammation and a-SMA protein expression. It was shown that chronic inflammation leads to increased expression of a-SMA protein, which activated hepatic stellate cells, leading to collagen deposition and consequently fibrosis in liver tissue (Ren et al., 2019). Liver fibrosis is an intermediate step in the further development of cirrhosis (Xu et al., 2021). This suggests that EcN-MT might reduce the production of chronic inflammation in the liver, thereby reducing the production of fibrosis and thus avoiding the development of cirrhosis. In addition, the results of oxidative stress factor assay showed that Cd exposure increased MDA and decreased CAT, SOD, and GSH expression, while EcN-MT treatment reduced MDA and increased CAT, SOD, and GSH. Some studies have demonstrated that Cd exposure caused excessive production of free radicals, which were responsible for oxidative stress in the body (Mezynska et al., 2018). Excess reactive oxygen species (ROS) could lead to an increase in MDA content by β-breakage of lipoxygenated lipids. To reduce oxidative stress, the body produced antioxidant enzymes such as SOD, CAT and non-enzymatic antioxidants (GSH) to maintain the dynamic balance of free radicals in the body (Moradkhani et al., 2020). This suggested that EcN-MT could reduce hepatic oxidative stress and thus alleviate Cd -mediated hepatotoxicity (Figure 2, Supplementary Figure S2).

To further investigate the potential mechanisms of EcN-MT to avoid Cd-induced subchronic liver injury, the TLR4/NF-κB inflammatory signaling pathway and key Bcl-2 family proteins were investigated. Although *in vitro* cellular assays were not used to validate the effect of cadmium exposure on the TLR4/NF-κB signaling pathway, however, many studies have shown that TLR4/NF-κB signaling pathway is closely associated with liver injury (Dapito et al., 2012; Arab-Nozari et al., 2020). The western blotting results showed that EcN-MT treatment significantly inhibited the expression of TLR4, which in turn downregulated MyD88, an intracellular linker protein downstream of TLR4, and thus inhibited NF-κB expression. It is well known that the conventional NF-κB is a heterodimer composed of p50 and p65 subunits, of which p65 is frequently detected by Western blotting (Zhang et al., 2018). Activated p50-P65 heterodimers translocate from the cytoplasm to the nucleus, triggering inflammation, promoting oxidative stress and participating in the *trans*-activation of various genes, such as apoptosis-related genes Bax and Bcl-2 (Michio Tamatani et al., 1999). Bcl-2 and Bax are members of the Bcl-2 gene, and Bcl-2 forms an unphosphorylated complex with Bax, so that its phosphorylation releases Bax from the Bcl-2-Bax complex and thus promotes cellular apoptosis (Karna et al., 2009). Meanwhile, NF-κB could play a transcriptional regulatory role to activate the expression of inflammatory genes such as IL-1β, IL-6 and TNF-α, which ultimately caused the release of inflammatory factors (Calisto et al., 2016). The results of inflammatory factor assay showed that supplementation with EcN-MT reduced the transcriptional levels of IL-1β, IL-6 and TNF-α inflammatory factors (Figure 3). In addition, according to the report (Chen et al., 2020), PDTC is the most commonly used inhibitor of NF-κB. Inhibition of NF-κB activation with PDTC also reduced inflammation, oxidative stress in the liver (Figure 5). This further suggested that EcN-MT reduced liver injury from Cd exposure possibly by inhibiting the activation of TLR4/NF-κB signaling pathway. More importantly, EcN treatment alleviated Cd expose-induced inflammation and oxidative stress in liver tissues to some extent, but not significantly, whereas MT and EcN-MT treatments effectively reduced Cd expose-induced inflammation and oxidative stress induced by Cd exposure. This suggested that EcN-MT suppressed the activation of TLR4/NF-κB signaling pathway mainly through expressed MT, thereby reducing liver injury.

Due to the direct anatomical link between the gut and liver and the recent rise of 16S rRNA sequencing technology, the relationship between gut microbiota and liver injury has been further explored. The results of Shannon index in α diversity and PCoA in β diversity indicated that EcN-MT appeared to promote the conversion of the intestinal microbiota of Cd-exposure mice to normal control mice. And then, the top 10 family level and genus level species composition results showed that supplementation with EcN-MT increased probiotic abundance including Ruminococcaceae at family level, *Akkermansia*, *Muribaculaceae*, *Lachnospiraceae* at genus level. Among them, Muribaculaceae belongs to the phylum Bacillus and could protect the organism through degraded dietary carbohydrates and antagonizing benzoates (Lagkouvardos et al., 2019). Likewise, Akkermania was discovered by scientists in 2004, which used intestinal mucin as an energy source to protect the intestinal tract from pathogens through competition, and the abundance of Akkermania was reduced in the intestinal microbiota of cadmium-exposed mice (Li et al., 2019). Also, recent studies have shown that Lachnospiraceae and Ruminococcaceae reduced *C. difficile* infections through the production of short-chain fatty acids that repressed pathogen proliferation and reduced intestinal inflammation (Collins et al., 2015; Guo et al., 2021). In brief, the increased abundance of intestinal probiotics leads to an increase in the production of short-chain fatty acids and an inhibition of pathogen proliferation, thereby reducing intestinal inflammation, which in turn reduces liver inflammation *via* the enterohepatic axis. This may be one of the important mechanisms by which EcN-MT treatment attenuates subchronic liver injury induced by Cd exposure (Figure 4). In addition, supplementation with EcN but not MT similarly ameliorated the intestinal microbiota disorder induced by Cd exposure, which suggests that EcN-MT may regulate the intestinal microbiota through the probiotic properties of the host bacterium EcN. Meanwhile, inhibition of NF-κB activation by PDTC treatment did not improve intestinal microbiome disorders (Figure 6), further suggesting that another mechanism of ECN-MT in the treatment of chronic Cd exposure is regulating intestinal microbiome through host bacteria EcN.

In summary, this study showed that EcN-MT treatment inhibited Cdexposure-induced liver injury by expressing MT, thereby increasing Cd excretion in the feces, reducing Cd accumulation in the liver, inhibiting TLR4/NF-κB activation, and reducing apoptosis and inflammatory factor transcription. In addition, EcN-MT treatment increased the abundance of probiotics and biodiversity through the host bacteria EcN, which transformed microorganisms ecology affected by Cd exposure to normal levels. Our experiment is the first to explore the potential role of EcN-MT in treating subchronic liver injury caused by cadmium exposure. Although this study only used mice for validation and nor other animals such as crab-eating monkeys because of economic and experimental conditions. However, the good therapeutic effect of engineered bacteria on liver injury has been proven, and with the development of synthetic biology and the further exploration of the role of probiotics in human health, it will provide a new therapeutic strategy for clinical treatment.

## Data Availability

The datasets presented in this study can be found in online repositories. The names of the repository/repositories and accession number(s) can be found below: NCBI Sequence Read Archive, BioProject ID PRJNA781077.
