# Supplementary material for: Engineered Bacteria EcN-MT Alleviate Liver Injury in Cadmium-Exposed Mice via its Probiotics Characteristics and Expressing of Metallothionein
Source: Front Pharmacol. 2022 Feb 24;13:857869. doi: 10.3389/fphar.2022.857869 (PMC8908209; doi:10.3389/fphar.2022.857869)

Supplementary Table 1

protein	Product Catalog	brand	dilution ratio
GAPDH	5174	Cell Signaling Technology	1:5000
a-SMA	14395-1-AP	Proteintech	1:2000
β-actin	66009-1-Ig	Proteintech	1:5000
TLR4	19811-1-AP	Proteintech	1:2000
MyD88	66660-1-Ig	Proteintech	1:5000
NF-κB	10745-1-AP	Proteintech	1:2000
p-NF-κB	AF2006	Affinity	1:2000
Bax	50599-2-Ig	Proteintech	1:5000
Bcl-2	12789-1-AP	Proteintech	1:5000
Cleaved Caspase-3	9661S	Cell Signaling Technology	1:1000
Occludin	27260-1-AP	Proteintech	1:5000

Supplementary Table 2

cytokines	forward prime Sequences	reverse prime Sequences
GAPDH	5′-AGCCAAAAGGGTCATCATCT-3′	5′-GGGGCCATCCACAGTCTTCT-3′
IL-1β	5′-GTGTCTTTCCCGTGGACCTTC-3′	5′-TCATCTCGGAGCCTGTAGTGC-3′
IL-6	5′- GAAATCGTGGAAATGAG-3′	5′-GCTTAGGCATAACGCACT-3′
TNF α	5′- GTGGAACTGGCAGAAGAGGCA-3′	5′-AGAGGGAGGCCATTTGGGAAC-3′

Supplementary Fig. 1

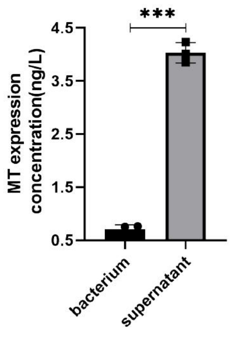

Supplementary Fig. 2

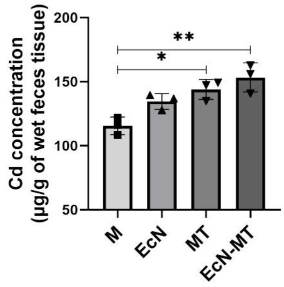

Supplementary Fig. 3

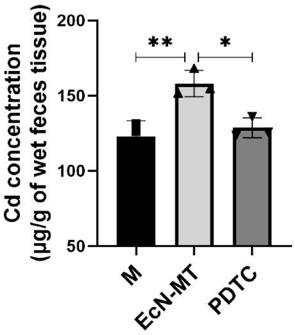

Supplement: Supplementary file 1 [file DataSheet1.pdf]
